# A 20-Year-Old Gossypiboma Causing Small Bowel Obstruction

**DOI:** 10.7759/cureus.36166

**Published:** 2023-03-15

**Authors:** Rema Alrashed, Hussam AlHarbi, Basmah A Alanazi, Mohammed Binaskar, Ibrahim Al Hasan, Abdullah A Algarni, Helayel Almodhaiberi

**Affiliations:** 1 General Surgery, Prince Sultan Military Medical City, Riyadh, SAU; 2 General Surgery, Al Kharj Armed Forces Hospital, Riyadh, SAU

**Keywords:** retained foreign body, abdominal pain, small bowel obstruction, exploratory laparotomy, abdominal surgery, surgical sponges, gossypiboma

## Abstract

Retained foreign bodies including gossypiboma could be silent for years. However, in some cases, it can lead to major complications. Gossypiboma is not frequently reported for multiple reasons, including nonspecific presentation clinically and radiologically, and ethical issues. We present a case of a gossypiboma that was retained for more than 20 years causing a severe intestinal obstruction for an elderly female. The intestinal obstruction was initially thought to be adhesive in nature and was managed initially conservatively, but with failure to improve, the patient was taken for exploratory laparotomy, and the foreign body was found attached to the root of the mesentery posterior to the transverse colon. This case sheds light on the fact that although surgical tools are of great utility, they must be managed with utmost care to prevent complications and secure patients’ safety.

## Introduction

Surgical sponges are essential tools in surgical procedures. When those sponges are retained in the body after closing the surgical site, an interaction between the immune system and the retained foreign body (RFB) may ensue. This inflammatory reaction will form gossypiboma, which might lead to significant morbidity and mortality [1]. Although operating rooms and surgeries are designed to function and be done in a sophisticated tightly controlled manner, mistakes happen, and the incidence of RFB may reach to one in 5,000 [1]. Radiological investigations such as ultrasound (US), computed tomography (CT), and magnetic resonance imaging (MRI) can aid in the diagnosis. Once diagnosed, surgical removal is advised for management. Gossypiboma should be considered a differential diagnosis in surgical patients presenting with a picture of bowel obstruction [2]. In this article, we present a case of a female in her eighth decade who had an RFB in her abdomen for 20 years before causing a major complication that required surgical management.

## Case presentation

A 71-year-old Saudi comorbid female presented to our emergency department with progressive, severe, and crampy abdominal pain for five days. The pain was constant, non-radiating, and associated with nausea and vomiting of food content multiple times. The patient gave a history of on-and-off attacks of the same pain for years; however, these attacks were less in severity and were relieved by laxatives at home. She is known to have atrial fibrillation and was on warfarin; she also had hypertension and diabetes mellitus. Her surgical history was significant for open cholecystectomy through a right paramedian incision 20 years ago and obstetric surgery 25 years back outside our hospital with unknown indication.

Upon examination, the patient was conscious, alert, oriented, and afebrile. She was not dehydrated or in pain. She was hemodynamically stable. Her abdomen was distended with scars from previous surgeries. She had generalized mild tenderness with no peritoneal signs. All hernial orifices were intact. Laboratory investigations were done for her (Table 1).

**Table 1:** Laboratory investigations upon presentation WBC: white blood count, HBG: hemoglobin, PLT: platelets, INR: international normalized ratio, Cr: creatinine, BUN: blood urea nitrogen, Na: sodium, K: potassium, ALT: alanine aminotransferase, ALP: alkaline phosphatase, CEA: carcinoembryonic antigen, CA 19-9: carbohydrate antigen 19-9, AFP: alpha-fetoprotein

Laboratory investigation	Result	Reference range
WBC	9.3 × 10^9^/L (81.8% neutrophils)	4-11 × 10^9^/L
HBG	12 g/dL	11-14 g/dL
PLT	242 × 10^9^/L	150-450 × 10^9^/L
INR	1.2	0.9-1.3
Cr	36 mcmol/L	45-84 mcmol/L
BUN	1.8 mmol/L	2.8-8.1 mmol/L
Na	139 mmol/L	136-145 mmol/L
K	3.7 mmol/L	3.5-5.1 mmol/L
ALT	15 unit/L	0-33 unit/L
ALP	82 unit/L	35-104 unit/L
Total bilirubin	6 mcmol/L	2-21 mcmol/L
CEA	4.2 ng/mL	0-2.5 ng/mL
CA 19-9	25 U/mL	0-37 U/mL
AFP	2.3 ng/mL	0-40 ng/mL

**Figure 1:**
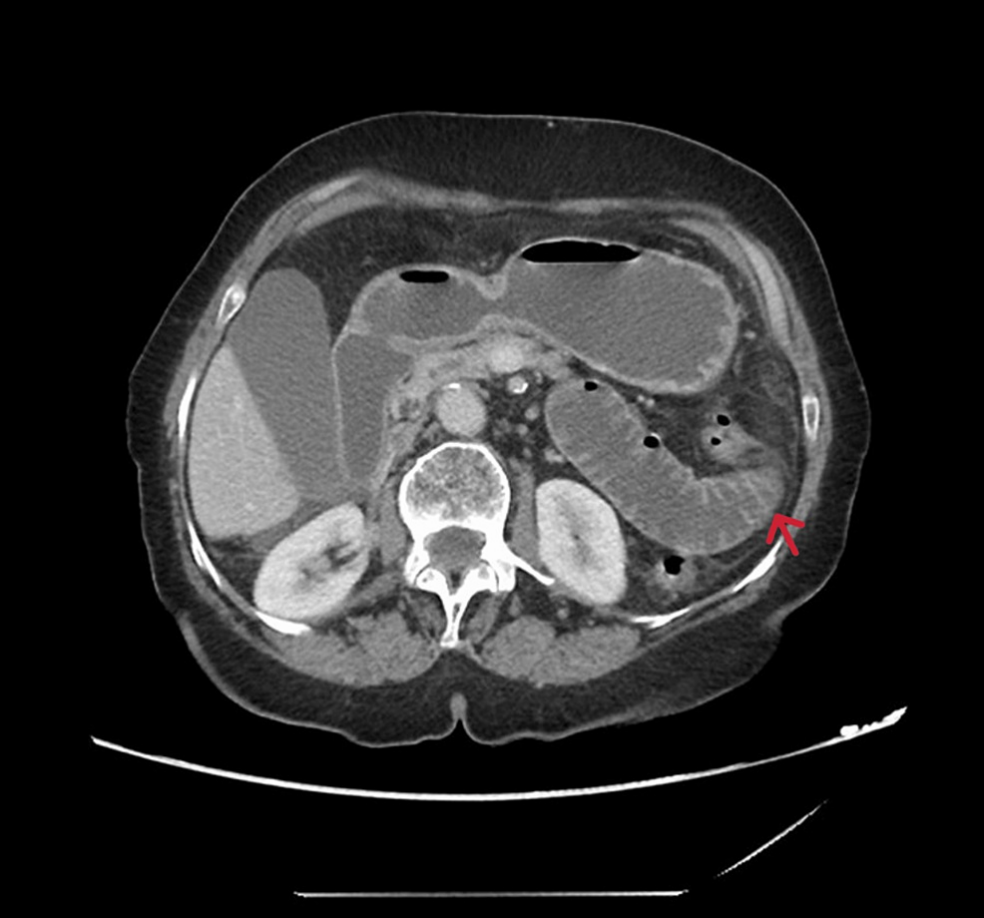
Abdominal CT in axial view showing focal obstruction with the transitional zone at the distal jejunal loop (red arrow) CT: computed tomography

**Figure 2:**
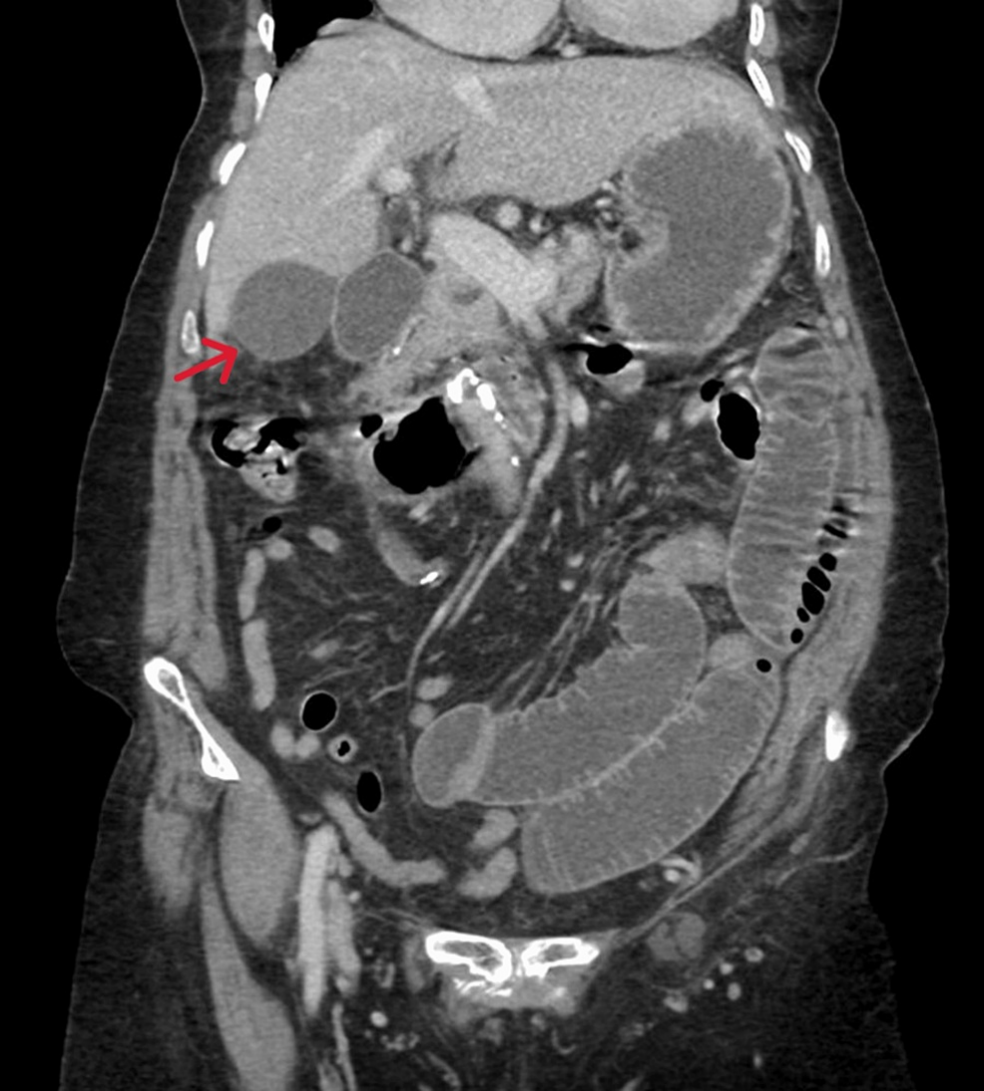
Abdominal CT in coronal view showing right subhepatic space cystic structure (red arrow) CT: computed tomography

On radiological investigation, an abdominal X-ray revealed a slightly prominent bowel loop with no significant air-fluid level, and there was no air under the diaphragm nor calcific densities noticed at the kidneys, ureter, or bladder region. Abdominal CT scan showed focal narrowing and obstruction with the transitional zone along the distal jejunal loop without associated local complication, with indeterminate mesenteric and right subhepatic space cystic lesion associated with calcification (Figure 1 and Figure 2).

The patient was admitted as a case of adhesive small bowel obstruction and managed initially conservatively. The patient improved clinically over the following days. She passed bowel motion on the second day of her admission; the diet was gradually advanced to a full liquid diet. On the sixth day of admission, she again suffered from crampy abdominal pain associated with nausea and constipation, and her general condition deteriorated. An abdominal CT scan was repeated and showed a high-grade small bowel obstruction, likely adhesive, with an interval increase in the inter-loop fluid and free fluid.

The patient was taken to the operating room. Laparotomy was done, and the intraoperative findings were ascites, multiple adhesions, and a hard mass with an irregular shape attached to the root of the mesentery, inferior to the transverse colon. The mass was opened during dissection, releasing calcified thick material. The mass was resected completely and sent to the histopathology laboratory.

The postoperative course was uneventful, and the patient was discharged with a follow-up in the clinic after two weeks. The final histopathology report was compatible with foreign material with secondary fibrosis, mixed inflammation, and calcification measuring 9 × 8 × 2 cm.

## Discussion

Gossypiboma and RFBs might result in morbidity, mortality, and medicolegal issues. RFB has an incidence of one in 1,000 abdominal surgeries; however, this number is not reliable due to the decrease in reporting [3].

A history of abdominal pain, constipation, nausea, vomiting, and/or fever in a patient with surgical history should raise suspicion of RFB. Abdominal CT can facilitate the diagnosis of RFB or gossypiboma. Radiological features may include mass with a hyperdense center [2].

Intraoperative radiographs can be misleading and could miss foreign bodies in up to 30% of patients, as the radiopaque marker of the surgical tools (such as gauze) can be twisted and can be confused with clips [4]. Postoperatively, radiographs may also still be misleading due to the fact that RFB can induce hematoma, fibrosis, cysts, abscesses, and calcifications [5].

After the diagnosis is made, RFB can be managed with open exploration and removal as it is the usual treatment [6]. The removal of RFB through the same surgical incision is recommended. However, the removal can be challenging as the retained sponge can lead to reaction and adherence to other structures. This has possible complications, including perforation of adherent structures and needle prick to the surgeon in case the RFB is sharp in nature [2].

Gawande et al. [7] and Lata et al. [5] in their work concluded that emergency surgery, unplanned change in the operation, and high body mass index (BMI) were significant risk factors for RFB, and a recommendation to radiographically screen high-risk patients was proposed by Gawande et al. [7]. Moreover, Lata et al. [5] in their work added miscommunication, improper gauze count, few staff members, and untrained staff as other risk factors for RFB [5].

The prevention of gossypiboma is crucial and can be achieved simply by pre- and postoperative count of surgical instruments and tools. Moreover, exploring the abdomen for any suspected RFB would help in preventing morbidity and mortality caused by RFB. It is worth mentioning that there are new preventive modalities that might decrease the incidence of RFB, such as electronically tagged sponges and electronic gauze counting [8].

## Conclusions

RFB is an infrequent but devastating complication of surgical procedures. It can lead to increased harm to both patients and healthcare workers. Teamwork and proper communication are key to avoiding such preventable medical errors. Multiple techniques and modalities were introduced that might help in reducing the incidence of RFB, such as electronically tagged sponges and electronic gauze counting; however, more studies are needed to evaluate their efficacy.
